# Short form scale of parent self-efficacy for general and specialized care for children with special health care needs

**DOI:** 10.1186/s41687-026-01143-9

**Published:** 2026-07-14

**Authors:** Carolyn Foster, Leonardo Barrera, Aaron Kaat, David Cella, Courtney Blackwell, Matthew Davis, Ryan Coller, Marie Heffernan

**Affiliations:** 1 Ann & Robert H. Lurie Children’s Hospital of Chicago, Chicago, USA; 2 Feinberg School of Medicine, Northwestern University, Chicago, USA; 3 Nemours Children’s Health System, Wilmington, USA; 4 Mayo Clinic Alix School of Medicine, Jacksonville, USA; 5 University of Wisconsin–Madison, Madison, USA

## Abstract

**Background:**

Self-efficacy in managing healthcare tasks has been shown to predict health behaviors and outcomes. Previous research has established the content validity of a full-length 30-item survey scale to assess the level of parents’ self-efficacy when managing their children’s healthcare tasks. The goal of this work was to create an efficient and psychometrically strong short form version to aid research and clinical practice.

**Methodology:**

We conducted scale construction using national cross-sectional survey data of English- and Spanish-speaking parents of children and youth with special health care needs (CYSHCN) 0–17 years old (9/2023-12/2023). Self-efficacy was assessed with a 5-point confidence scale [1 (not confident) to 5 (very confident)]. The sample was randomly evenly split into derivation and validation samples. Exploratory factor analyses (EFAs) and confirmatory factor analyses (CFA) were performed for the general (14-item), equipment (8-item), and medication care (6-item) long form subscales, but not the therapy and mobility care long form subscales, which were only 1-item long. Spearman correlations were used to assess convergent and divergent validity. Means and standard deviations (SD) were calculated for item scoring.

**Results:**

1,132 respondents participated (54% Non-Hispanic White; 68% mothers). Derivation and validation samples were *n* = 566 each. The EFAs revealed single-factor structures for general care items (Cronbach’s alpha 0.95); medication care items (Cronbach’s alpha 0.91); and equipment care items (Cronbach’s alpha 0.85). CFA results confirmed EFA results, with 5 items for general care (Cronbach’s alpha 0.87), 3 items for medication care (Cronbach’s alpha 0.76), and 3 items for equipment care (Cronbach’s alpha 0.77). The mean scores of the new short form subscales were 4.36 (SD = 0.73), 4.41 (SD = 0.77), and 4.19 (SD = 0.86) for general, medication, and equipment care, respectively.

**Discussion:**

This 13-item short form scale (5 general care, 3 equipment care, 3 medication care, 1 therapy care, and 1 mobility care sub-scales items) is an efficient, self-administered means of assessing parents of CYSHCN’s self-efficacy with managing their child’s healthcare. Future work will test the scale’s feasibility and utility at identifying parents who may benefit from additional support and training.

**Supplementary Information:**

The online version contains supplementary material available at https://doi.org/10.1186/s41687-026-01143-9.

## Introduction

Self-efficacy, defined as one’s confidence in completing a task, has been shown to be an important component of health behaviors, daily living skills, and self- and family-delivered medical care both in adults and children [1–10]. Higher self-efficacy by family caregivers in managing their children’s healthcare tasks, such as giving medication or scheduling appointments, has been associated with better health outcomes in several pediatric chronic conditions [2–10]. 

Self-efficacy related to medical caregiving is especially relevant for children and youth with special healthcare needs (CYSHCN), defined as children who require healthcare beyond what an otherwise healthy child would require in order to treat a physical, developmental, behavioral, or emotional condition [11]. With the rising prevalence of CYSHCN with rare, fragile, and/or disabling health conditions, there has been growing recognition that family caregivers must learn to master increasingly complex healthcare tasks at home [12, 13]. Examples of children with medical complexity’s (CMC’s) home care tasks include sterile dressing changes, liquid medicine preparation and administration through varied routes, and medical equipment maintenance. The ability to measure a family caregiver’s self-efficacy to carry out these and other tasks may provide an opportunity to identify family caregivers in need of improved support prior to discharge home or over the course of their child’s healthcare journey. However, there is a dearth of validated person-reported outcome measures (PROMs) to measure and track family caregiver levels of self-efficacy in managing pediatric healthcare tasks across a range of conditions [8, 14]. 

To address this gap, a condition-agnostic 30-item PROM was developed to measure family caregiver self-efficacy of managing child health conditions for family caregivers who identify as taking care of a CYSHCN [2]. This full-length scale was adapted from the National Institutes of Health funded Patient-Reported Outcomes Measurement Information System^®^ (PROMIS^®^) adult self-efficacy instrument [15, 16], and pediatric content was adapted using expert ranking (including caregivers) and cognitive interviews in English- and Spanish-speaking caregivers of CYSHCN [2]. 

While the full-length self-efficacy scale has the potential for identifying family caregivers with low self-efficacy across a range of healthcare tasks, the scale had never been optimized in a large sample. Further, its current length of 30 items raised concerns that the time to complete the full-length scale could limit its use in busy clinical settings or research project when accompanied by other scales [17, 18]. Therefore, the goals of the current study were to create an efficient and psychometrically strong tool to aid research and clinical practice among families with CYSHCN, as has been done in other research areas with families [19]. 

## Methods

We engaged in a multi-step scale construction process [20]. Survey item generation was published previously, including content validation and initial testing [2]. Presented here are the subsequent steps including scale administration and item selection followed by subscale evaluation.

### Item generation

The full questionnaire includes 30 items, rated on a 5-point scale [(1) I am not confident at all; (2) I am a little confident; (3) I am somewhat confident; (4) I am quite confident; (5) I am very confident], with two section [2]. The first section includes items about self-efficacy for general care that were broadly applicable to all parents of CYSHCN (14 items). The second section includes items that were about self-efficacy for specialized care, including: medication (8 items), equipment (6 items), therapy exercises (1 item), and moving the child (1 item). Given that not all CYSHCN use medication, equipment, therapy or mobility supports (respectively), the second section items are administered only if the parent identified that their child takes medicine on a regular basis, required specialized equipment, engages in therapy such as physical therapy, and needs help moving around. An introductory question for each of these survey sections was used to determine whether those items were relevant to the respondent’s children (e.g. Does your child take any medications on a regular basis?). If the respondent indicates that the section was relevant to their child, then they receive all items in that section. For all self-efficacy items, scoring was as follows: 1 = not confident, 2 = a little confident, 3 = somewhat confident, 4 = quite confident, 5 = very confident. Higher mean scores for a subscale indicated higher sense of self-efficacy on that subscale.

### Scale administration and item selection

Next, we administered the full 30 item scale to a national sample of U.S. parents in English and Spanish via a double-opt in QualtricsXM research panel between September 8, 2023 – December 6, 2023. Parents were eligible for the survey if they had a CYSHCN who was between the ages of 0–17 years old using the standard National Survey for Children’s Health screener developed by the Maternal Child Health Bureau, which includes questions that cover medication use, functional impairment, and healthcare use [21]. Quota sampling was used to obtain a sample of parents that resembled the U.S. population for race and ethnicity. The electronic survey invitation was sent through the QualtricsXM dashboard. Once a quota was filled, potential respondents who fit that quota were no longer able to complete the survey. The survey contained items on related topics that are outside the scope of the current project, including perceptions and beliefs about immunization and school attendance.

Parents were permitted to skip items. Qualtrics compensated using its points-based rewards system, whereby participants earned points for completing the survey, and after accruing a certain number of points, they can exchange them for rewards such as gift cards. This study was determined to be exempt from human subjects’ regulation, in accordance with the Declaration of Helsinki, as it analyzed de-identified panel data (Anonymized). Qualtrics maintained sufficient identifiable information to confirm a survey was completed by only one induvial and has internal procedures for quality responses, including speed checks for quality responses (i.e. anyone finishing < 1/3 median time of 9 min was removed and replaced). To further confirm the data quality, prior to analyses, our team further checked to ensure that the inclusion criteria had been met (i.e., child age, answers to screener). Missingness and distributions of all variables were assessed for concerning trends and we confirmed data completeness and response descriptions matched our expectations. Skip logic was confirmed to not be violated.

We randomly split the sample evenly into a derivation sample and a validation sample [22]. The derivation sample was used for exploratory factor analysis (EFAs) and for item reduction to create a preferred short form. The validation sample was used to cross-validate the optimal structure for the suggested short form via confirmatory factor analysis (CFAs).

#### Exploratory factor analyses

To create a short-from that reflected the response logic, we conducted three unweighted least squares EFAs on the subscales from the original full-form scale: one for the self-efficacy for general care subscale (14 items), one for the self-efficacy for medication care subscale (8 items), and one for the equipment care subscale (6 items). However, we did not attempt to further shorten the therapy and mobility care sections as they already consisted of single items within the full-scale form.

We used a variety of criteria to determine the optimal number of factors to extract within each of the item subsets, including the Kaiser criterion (i.e., number of eigenvalues greater than or equal to 1) [23, 24], the scree plot [25], and the ratio of the first and second eigenvalues. The number of eigenvalues above the “elbow” in a scree plot is typically considered the optimal number of factors. A ratio of the first to second eigenvalue greater than four tends to support unidimensionality [26]. We also examined item loadings and internal consistency reliability (Cronbach’s alpha) to select short form items for the three subscales: general caregiver self-efficacy, medication self-efficacy, and equipment self-efficacy. Factor analyses were conducted in SPSS. Mean substitution was used for missing values and items were treated as ordinal. We reviewed the item loadings on each factor. Items that loaded 0.40 or higher were considered to load on a particular factor [27]. 

#### Item selection

Criteria that we used to select items included: factor loadings with higher loadings indicating a preferred item; Cronbach’s alpha if item deleted, with a higher value if item deleted indicating less desirable item; item-total correlation; percent of respondents who scored “lower self-efficacy” (those who indicated they felt “not confident,” “a little confident,” or “somewhat confident”) and the item mean score. We included these last two criteria because we wanted to select items that ensured a broad range of responses and minimized ceiling effects. For the general care self-efficacy subscale, we aimed to select no more than five items for the short form; for the two specialized subscales (medication care self-efficacy and equipment care self-efficacy), we aimed to select no more than four items for the short forms. This was done to keep the short forms manageable and practicable for administration in time-constrained, in-person, clinical settings.

### Short form evaluation

After conducting the three EFAs and selecting items for the three short form subscales, we used the validation sample to conduct three CFAs, one for each subscale, to confirm factor structure of the short form subscales. We conducted three CFAs, one for each subscale: the self-efficacy for general care subscale, one for medication care subscale, and one for equipment care subscale.

We cross-validated the structures proposed by the EFAs within a CFA context, using the same criteria to evaluate factor loadings and eigenvalues. With the validation sample, we also assessed internal consistency reliability with Cronbach’s alpha, which were computed for each subscale. We assessed model fit for the CFAs using multiple fit indices. First, we used the 𝜒^2^ for overall model fit. However this test is known to be sensitive to large sample sizes, and can indicate poor model fit (i.e., a low p-value), even when there is only a trivial misfit [28]. Therefore, we followed guidance put forth by Alavi and colleagues (2020) to provide a more holistic view of goodness of fit, and included the root mean squared error of approximation (RMSEA) for absolute fit, and the comparative fit index (CFI) and Tucker Lewis Index (TLI) for incremental fit [29, 30]. 

We used Spearman correlations to assess convergent and divergent validity of the short form. We computed correlations of the short form subscales with each other, with the full-length form, and with the remaining items on each full-length form after removing the short form items. We also computed correlations of the short form subscales with demographic measures (years caring for the child with special healthcare needs, parent education level) to explore whether there were associations with these variables. We expected that parents with more years caring and with higher education level would have higher levels of self-efficacy related to their child’s care, however we did not have an a priori hypothesis about the magnitude of these correlations.

## Results

In total, 1,132 parents who had CYSHCN completed the survey. In terms of the health status of the children in the surveyed families, 19.3% were in excellent health, 28.0% very good, 29.0% good, 19.1% fair, and 4.7% poor health, per parent report. We also assessed characteristics of the children’s healthcare needs: 79.9% required medicine prescribed by a doctor,;71.6% required more medical care, mental health or educational services than is usual for most children their age; 47.4% were limited in their ability to do things other children of the same age can do; 49.0% received special therapy, such as physical, occupational, or speech therapy; and 61.7% had an emotional, developmental, or behavioral problem for which they receive treatment or counseling.

The analytic sample was randomly split into a derivation sample (*n* = 566) and a validation sample (*n* = 566). In the derivation sample, 54.2% of parents were white and in the validation sample, 60.4% of parents were white (*p* = 0.21) (Table 1). Most respondents were a mother figure to the CYSHCN (68.6% in the derivation sample, 64.1% in the validation sample, *p* = 0.23), the average parent age was 38.8 years (*SD* = 10.0) in the derivation sample and 38.2 years (*SD* = 9.2) in the validation sample (*p* = 0.28). In both samples, over half of the children were older than 10 years (50.4% in the derivation sample; 51.1% in the validation sample, *p* = 0.94).

**Table 1:** Parental respondent and child demographic information

Parent Demographic Information	Derivation Sample for Exploratory Factor Analysis (*n* = 566)						Validation Sample for Confirmatory Factor Analysis (*n* = 566)					
	General Care (*n =* 566)		Medication Care (*n* = 416)		Equipment Care (*n* = 113)		General Care (*n* = 566)		Medication Care (*n* = 401)		Equipment Care (*n* = 137)	
	%	*n*	%	*n*	%	*n*	%	*n*	%	*n*	%	*n*
**Relationship to child**												
Mother figure	68.6	388	65.5	273	57.7	65	64.1	363	62.3	250	51.8	71
Father figure	26.1	148	29.1	121	38.9	44	28.8	163	30.7	123	41.6	57
Grandparent, legal guardian, or other	5.3	30	3.8	16	1.8	2	7.0	40	3.5	14	3.6	5
**Parent education**												
College degree or higher	37.6	213	39.9	166	47.8	54	40.6	230	44.9	180	55.5	76
Some college	33.2	188	32.0	133	23.9	27	29.2	165	29.4	118	18.2	25
High school or below	29.2	165	28.1	117	28.3	32	30.2	171	25.7	103	26.3	36
**Marital status**												
Married or in a partnership	64.5	365	67.1	279	65.5	74	65.9	373	67.3	270	73.0	100
Single or never married	18.4	104	14.2	59	18.6	21	20.3	115	17.7	71	17.5	24
Widowed, divorced, or separated	17.1	97	18.8	78	15.9	18	13.8	78	15.0	60	9.5	13
**Years Caregiving**												
Less than 6 months	8.1	46	6.7	28	6.2	7	7.6	43	7.0	28	12.4	17
6 months − 1 year	14.3	81	14.9	62	23.9	27	14.3	81	14.5	58	19.0	26
1–3 years	28.6	162	29.8	124	31.0	35	27.4	155	26.7	107	32.8	45
3–5 years	19.8	112	19.7	82	14.2	16	20.1	114	19.7	79	15.3	21
5–10 years	16.6	94	15.4	64	15.0	17	16.4	93	17.2	69	13.9	19
More than 10 years	12.5	71	13.5	56	9.7	11	14.1	80	15.0	60	6.6	9
**Survey Language**												
English	99.5	563	99.5	414	100	113	99.9	561	99.0	397	98.5	135
Spanish	0.5	3	0.5	2	0	0	0.9	5	1.0	4	1.5	2
	***M***	***SD***	***M***	***SD***	***M***	***SD***	***M***	***SD***	***M***	***SD***	***M***	***SD***
**Parent age**	38.8	10.0	39.4	10.2	36.7	9.7	38.2	9.2	38.5	9.2	34.6	7.8
**Child Demographic ** **Information**	**%**	***n***					**%**	***n***				
**Child race/ethnicity**												
Non-Hispanic White	54.2	307	58.2	242	48.7	55	60.4	342	62.6	251	56.9	78
Hispanic/Latinx	18.4	104	16.8	70	21.2	24	15.4	87	15.2	61	21.9	30
Black	13.6	77	12.3	51	22.1	25	11.8	67	10.7	43	10.2	14
Another race or ethnicity	13.8	78	12.7	53	8.0	9	12.4	70	11.5	46	10.9	15
**Child age**												
0–3 years	9.7	55	8.2	34	14.2	16	9.2	52	5.0	20	10.2	14
4–10 years	39.9	226	38.2	159	38.9	44	39.8	225	37.2	149	47.4	65
> 10 years	50.4	285	53.6	223	46.9	53	51.1	289	57.9	232	42.3	58
**Child sex assigned at birth**												
Male	56.9	322	55.3	230	57.5	65	55.5	314	55.9	224	54.7	75
Female	41.3	234	43.5	181	40.7	46	43.8	248	43.1	173	44.5	61
Non-binary/other/no response	1.7	10	1.2	5	1.8	2	0.7	4	0.9	4	0.7	1

### Exploratory factor analyses (EFA)

#### Self-efficacy for general care EFA

The EFA of the self-efficacy for general care items revealed a one-factor structure. There was one eigenvalue above Kaiser’s criterion and an eigenvalue ratio of 8.81, supporting a one-factor solution. The Cronbach’s alpha for all 14 items on the self-efficacy for general care full-length subscale was 0.95, indicating high internal consistency. All items loaded on the single factor at 0.59 or above. Table 2a shows all factors.

**Table 2:** Exploratory factor analysis results. Part a shows the factors for the general care items; part b shows results for medication care items; and part c shows results for equipment care items. Asterisked items were selected for short form inclusion

Item	Factor 1 Loading	Cronbach’s Alpha if Item Deleted	Item-Total Correlation	% with Lower self-efficacy	Item mean score
**a: Self-Efficacy General Care (** ***n*** ** = 566, Cronbach’s alpha = 0.95)**					
I can work with my child’s doctor(s) to choose the treatment that seems right for my child, including the option of not giving any treatment.*	0.78	0.94	0.78	19.78	4.32
I know when my child needs to be seen by a healthcare provider, if sick.*	0.78	0.94	0.78	13.39	4.48
I can actively participate in decisions about my child’s treatment.	0.76	0.94	0.76	15.30	4.48
I know what to do if my child’s misses a medication or other type of treatment.*	0.74	0.94	0.75	18.94	4.36
I can tell when my child’s symptoms worsen.	0.73	0.94	0.75	14.84	4.43
I can follow my child’s full treatment plan (including medication, therapy, and other care)	0.73	0.94	0.75	14.75	4.48
I can find information to learn more about my child’s treatment.	0.72	0.94	0.71	15.10	4.45
I know the steps needed to schedule my child’s healthcare appointments.*	0.72	0.94	0.76	11.68	4.55
I know the steps needed to get in contact with my child’s healthcare providers when I have a question or concern about my child’s care.	0.72	0.94	0.72	15.75	4.46
I can follow my child’s diet or feeding plan.	0.71	0.94	0.73	15.14	4.47
I can figure out what my child needs when my child’s symptoms change.	0.71	0.94	0.71	21.02	4.24
I know what to do if my child has a medical emergency until help arrives.*	0.69	0.94	0.70	21.17	4.27
I know the healthcare condition(s) that affect my child.	0.69	0.94	0.70	23.64	4.24
I know how to set up a telemedicine video visit for my child using an electronic device.	0.59	0.95	0.60	21.39	4.27
**b: Self-Efficacy for Medication Care (*n*** **= 416**,** Cronbach’s alpha = 0.91)**					
I can give my child’s medications when they are scheduled to be given.	0.82	0.90	0.79	12.22	4.53
I know how to give my child’s medications (such as by mouth or by tube).*	0.81	0.90	0.79	11.79	4.58
I know how to arrange for my child to receive medication or treatments at locations other than home, if needed.*	0.75	0.90	0.72	14.81	4.42
I know how to get my child’s medication refilled, if it is needed.	0.76	0.90	0.73	9.27	4.59
I can continue my child’s medications or other treatment when we are away from home.	0.73	0.90	0.71	15.42	4.47
I can keep a list my child’s medication, including the medication doses and schedule.	0.72	0.91	0.70	15.31	4.48
I can tell the difference between when my child is having a medication side effect or experiencing symptoms of their condition(s).*	0.69	0.91	0.67	23.65	4.22
I know what to do when my child’s medication refill seems different than usual.	0.67	0.91	0.67	15.12	4.42
**c: Self-Efficacy for Equipment Care (*n*** **= 113**,** Cronbach’s alpha = 0.85)**					
I can change my child’s disposable supplies or devices (such as diabetes pump, tracheostomy, line dressing, etc.).*	0.72	0.82	0.71	21.70	4.31
I can use my child’s medical equipment by myself.	0.74	0.84	0.65	22.52	4.18
I can tell when parts of my child’s medical equipment, parts, or supplies needs to be replaced or repaired.*	0.67	0.82	0.68	25.00	4.19
I can use my child’s mobility equipment, such as a wheelchair, walker, or lift.	0.64	0.84	0.61	12.90	4.32
I can clean my child’s medical equipment.	0.63	0.84	0.57	17.92	4.34
I know the settings on my child’s medical equipment (such as a pump, monitor, ventilator, etc.).*	0.67	0.82	0.68	18.52	4.21

Note. “Lower self-efficacy” was defined as those who reported feeling “not confident,” “a little confident,” or “somewhat confident”

We selected items to retain on the short form self-efficacy for general care subscale based on several considerations, including factor loadings ( > = 0.40), and Cronbach’s alpha if item deleted (aiming for smaller reductions in Cronbach’s alpha from the original scale). We also examined the following: the item-total correlation (selected items with higher item-total correlation when possible); the percent of respondents who scored “lower self-efficacy” on each item (with the goal of selecting items that had a larger proportion of parents who scored “lower self-efficacy”); the item mean score (preference for items with lower mean scores to avoid ceiling effects); and content coverage. As an example, we selected the item, “I can work with my child’s doctor(s) to choose the treatment that seems right for my child, including the option of not giving any treatment” because this item had a high factor loading (0.78), the Cronbach’s alpha did not significantly improve when deleting the item, the item-total correlation was relatively high (0.78), there was a substantial proportion of parents who scored “low self-efficacy” for the item (19.78%), and the item mean score was relatively low compared to other items (*m* = 4.32). Alternatively, we omitted an item that assessed a similar aspect of being involved in the decisions about the child’s care (“I can actively participate in decisions about my child’s treatment”) because it had a slightly lower factor loading (0.76), lower proportion of parents who scored “low self-efficacy” (15.30%), and had a higher mean score (*m* = 4.48). In total, we selected five items for the short form subscale self-efficacy for general care, denoted by asterisks in Table 2a.

#### Self-efficacy for medication care EFA

The criteria also supported a single-factor structure for the self-efficacy for medication care items. There was one eigenvalue above Kaiser’s criterion and an eigenvalue ratio of 7.32, supporting a one-factor solution. The Cronbach’s alpha for the full-length 8-item self-efficacy for medication care subscale was 0.91, indicating high internal consistency. We used the same criteria for item selection as described for the self-efficacy for general care subscale. For instance, we selected the item, “I know how to give my child’s medications (such as by mouth or by tube)” (factor loading 0.81) rather than the similar item, “I can give my child’s medications when they are scheduled to be given” (factor loading 0.82) because the two items had similar factor loadings and performed similarly across the other criteria used for item selection, but the former had a broader scope (medication administration) than the latter (timing of medication administration). Item selection yielded three items selected for the short form for self-efficacy for medication care, denoted by asterisks in Table 2b.

#### Self-efficacy for equipment needs EFA

The EFA of the self-efficacy for equipment care items also revealed a one-factor structure, with one eigenvalue above Kaiser’s criterion and an eigenvalue ratio of 3.98. Although an eigenvalue ratio over 4 is often considered evidence of subscale unidimensionality, considering that this is a shorter subscale (only 6 items), and both the scree plot (see Supplemental Fig. 1) and the eigenvalue ratio suggests a single factor for self-efficacy for equipment care subscale, we proceeded with modeling this as a unidimensional construct. The Cronbach’s alpha for the full-length subscale of 6 items was 0.85, indicating high internal consistency. We used the same criteria for item selection as described for the self-efficacy for general care subscale. As an example, we selected the item, “I can tell when parts of my child’s medical equipment, parts, or supplies needs to be replaced or repaired.” This item had a factor loading of 0.67; the Cronbach’s alpha did not improve significantly if the item was deleted; the item-total correlation was relatively high (0.68); and importantly, there was a higher proportion of parents who scored “low self-efficacy” for this item (25%), and the mean was relatively low (*m* = 4.19). Item selection yielded three items for the short form for self-efficacy for equipment care subscale, denoted by asterisks in Table 2c.

### Confirmatory factor analysis (CFA)

For each short form subscale, we restricted the number of factors to one for the CFA (Table 3). For the general care subscale, all five items loaded on a single factor (0.66–0.70) with high internal consistency (Cronbach’s alpha = 0.87). The 𝜒^2^ test for overall model fit indicated somewhat poor fit (𝜒^2^ [5] = 13.049, *p* = 0.023). However, given the limitations of the 𝜒^2^ test for model fit with large sample sizes, we assessed other model fit indicators. The RMSEA indicated good absolute fit (0.054), and the incremental fit indices also indicated excellent fit (CFI = 0.99; TLI = 0.99). For the medical care subscale, all three items loaded onto one factor (0.68–0.72), showed acceptable internal consistency (Cronbach’s alpha = 0.76), and excellent model fit (𝜒^2^[0] = 294.345, *p* = 0.000; RMSEA = 0.000; CFI = 1.000; and TLI = 1.000). Finally, for the equipment needs subscale, the three items loaded onto one factor (0.71–0.82) that had acceptable internal consistency (Cronbach’s alpha = 0.77), and excellent model fit (𝜒^2^[0] = 90.186, *p* = 0.000; RMSEA = 0.000; CFI = 1.000; and TLI = 1.000) (Table 4a-c).

**Table 3:** Confirmatory factor analysis results. Parts a, b, and c show the results for the 5 general care items; 3 medication care items, and 3 equipment care items selected for the short form, respectively

Item	Factor 1 Loading	Cronbach’s Alpha if Item Deleted	Item-Total Correlation
**a: Self-Efficacy for General Care (** ***n*** ** = 566, Cronbach’s alpha = 0.87)**			
I can work with my child’s doctor(s) to choose the treatment that seems right for my child, including the option of not giving any treatment.	0.70	0.84	0.67
I know when my child needs to be seen by a healthcare provider, if sick.	0.79	0.83	0.73
I know what to do if my child’s misses a medication or other type of treatment.	0.79	0.83	0.72
I know the steps needed to schedule my child’s healthcare appointments.	0.76	0.83	0.71
I know what to do if my child has a medical emergency until help arrives.	0.66	0.86	0.62
**b: Self-Efficacy for Medication Care (*n*** **= 401**,** Cronbach’s alpha = 0.76)**			
I know how to give my child’s medications (such as by mouth or by tube).	0.72	0.68	0.60
I know how to arrange for my child to receive medication or treatments at locations other than home, if needed.	0.74	0.66	0.62
I can tell the difference between when my child is having a medication side effect or experiencing symptoms of their condition(s).	0.68	0.71	0.57
**c: Self-Efficacy for Equipment Care (*n*** **= 137**,** Cronbach’s alpha = 0.77)**			
I can change my child’s disposable supplies or devices (such as diabetes pump, tracheostomy, line dressing, etc.).	0.82	0.61	0.67
I can tell when parts of my child’s medical equipment, parts, or supplies needs to be replaced or repaired.	0.58	0.77	0.53
I know the settings on my child’s medical equipment (such as a pump, monitor, ventilator, etc.).	0.71	0.67	0.62

Note. “Lower self-efficacy” was defined as those who reported feeling “not confident,” “a little confident,” or “somewhat confident”

**Table 4:** Model fit indices for the confirmatory factor analysis

Model	Overall Model Fit		Absolute Fit	Incremental Fit	
	χ^2^ (df)	*p*	RMSEA	CFI	TLI
Self-Efficacy for General Care	1161.414 (5)	0.000	0.054	0.993	0.986
Self-Efficacy for Medical Care	294.345 (0)	0.000	0.000	1.000	1.000
Self-Efficacy for Equipment Care	90.186 (0)	0.000	0.000	1.000	1.000

#### Scale evaluation

The means, standard deviations (SD), and Cronbach’s alphas for the three short form subscales are presented in Table 5, which also includes the same information for the three full-length subscales for comparison. The means for all 3 short form subscale were very similar to the means for the full-length subscales. The Cronbach’s alphas for all three short forms were > 0.70, suggesting that the three short form subscales have sufficient reliability.

**Table 5:** Comparison of short-form and full-length scale

Self-Efficacy Subscales	*N*	Mean	SD	Cronbach’s alpha	*% Floor Effect*	% Ceiling Effect
**General Care**						
Short form (5 items)	563	4.36	0.73	0.87	0.4	31.4
Full length (14 items)	564	4.36	0.70	0.95	0.2	19.9
**Medication Care**						
Short form (3 items)	398	4.41	0.77	0.76	0.3	43.2
Full length (8 items)	400	4.47	0.70	0.91	0.3	34.3
**Equipment Care**						
Short form (3 items)	135	4.19	0.86	0.77	0.7	34.1
Full length (6 items)	136	4.26	0.76	0.85	0.7	27.9
**Therapy Care** (1 item)	233	4.28	1.02	--	0.4	60.1
**Mobility Care** (1 item)	86	4.26	1.17	--	3.5	65.1

Note. % Floor Effect = the proportion of respondents who had the lowest score on the subscale; % Ceiling Effect = the proportion of respondents who had the highest score on the subscale

Each of the three short form subscales also correlated highly with the full-length version of the corresponding full-length version (Table 6b). For instance, scores on the self-efficacy for equipment care short form and the full-length self-efficacy for equipment care subscale had a correlation of *r* = 0.93 (*p* < 0.01). The short form subscales were significantly correlated with one another, with the highest correlation between the general care and equipment care short forms (*r* = 0.76, *p* < 0.001) (Table 6a). The scores on the short form subscales also were correlated with scores on the remaining self-efficacy items that were not selected for the short form. For instance, the correlation between scores on the self-efficacy for general care short form and the remaining general care items that were not selected for the short form was *r* = 0.87 (*p* < 0.01). The correlations between the scores on the short form subscales and demographic variables reflected the same pattern of correlations between the full-length form and demographic variables, albeit the correlations with the short form subscales were slightly lower in magnitude. For instance, the correlation between the self-efficacy for medication care short form subscale and number of years caring was *r* = 0.25 (*p* < 0.01), whereas the correlation between the full-length self-efficacy for medication subscale and number of years caring was *r* = 0.29 (*p* < 0.01). The final short-form survey is included as an appendix.

**Table 6:** Correlation of short form items. Part a reports the correlations between the 3 short form subscales; part b reports the correlations of the short forms with the full-length measures; and part c reports the correlation of the short forms with other items within the administered survey

	Short Form Self-Efficacy Subscales		
	General Care (5-Item Short-Form)	Medication Care (3-Item Short Form)	Equipment Care (3-Item Short Form)
**a. Correlations with Full Length Measures**			
**General Care**			
** Full Subscale**: All General Care Items (14 items)	0.93**	0.73**	0.74**
** Remaining Items**: General Care (5 short form items removed)	0.84**	0.71**	0.72**
**Medication Care**			
** Full Subscale**: All Medication Care Items (8 total items)	0.75**	0.90**	0.74**
** Remaining Items**: Medication Care (3 short form items removed)	0.70**	0.72**	0.72**
**Equipment Care**			
** Full Subscale**: All Equipment Care (6 total items)	0.80**	0.75**	0.92**
** Remaining Items**: Equipment Care (3 short form items removed)	0.76**	0.72**	0.72**
**b. Correlations across Short Form Subscales**			
**Short Form Self-Efficacy Subscales**			
General Care (5-Item Short-Form)	--	--	--
Medication Care (3-Item Short Form)	0.70**	--	--
Equipment Care (3-Item Short Form)	0.73**	0.73**	--
**c. Correlations with Demographics and Other Measures**			
**Demographic Variables**			
Years Caring	0.29**	0.25**	0.30**
Parent Education Level	0.00	-0.03	0.1

**. Correlation is significant at the 0.01 level (2-tailed)

*. Correlation is significant at the 0.05 level (2-tailed)

## Discussion

This new short form is an efficient, self-administered means of assessing English and Spanish language family caregivers’ feelings of self-efficacy in their ability to manage their child with special health care needs’ healthcare tasks. All three new subscales demonstrate strong internal consistency, acceptable model fit, and correlate with the respective full-length form from which they are derived. Each short form subscale is intentionally brief, enabling their use in fast-paced clinical settings in which time is limited. It is important to note that not all caregivers received the self-efficacy for medication subscale or the self-efficacy for equipment subscale (i.e., if their child did not require medication or equipment), but all caregivers received the general self-efficacy subscale. Therefore, we conducted three separate factor analyses with one-factor solutions rather than a single factor analysis with a three-factor solution.

We note that aside from the general care subscale, all the specialized care subscales (medication, equipment, therapy, and mobility) are dependent on an individual child’s medical needs. By conducting analysis within subscales, this allows users of this survey to select which subscales are most relevant for their purposes. For example, a busy asthma or diabetes clinical program may benefit from primarily distributing the general, medication, and equipment care short form questions to quickly screen family caregivers for brief training intervention but have lower utility for therapy and mobility given the lower levels of physical impairment in those clinical populations. Conversely, a complex care program with a high proportion of patients with disabling conditions and longer visit slots may choose to administer all subscales in short or long form, potentially paired with longer training sessions to support equipment, therapy, and mobility tasks skills. Further evaluation of implementation of this short form into real-world settings will be needed to inform the ideal methods for its adoption and sustainability.

By calculating the means and SD of respondents in both the long and short form scales we also identified a potential approach to identify respondents who are statistically outside of the “typical” response among a national cohort of parents of CYSHCN. We note that validation of scoring will be needed to assess whether a “positive” screen – defined as a respondent who is 1 or 2 SD outside of the national cohort response – correlates with outcomes of interest including lower competency at tasks. However, this initial statistical scoring approach could provide clinicians with a method to identify respondents falling outside “typical” responses and who may benefit from further support and/or training. Notably, while CYSHCN is a widely studied group, it does cover a broad range of health conditions with varied levels of severity. Examining self-efficacy within sub-populations, for example those with medical complexity, may identify differing patterns of responses given differing levels of training and exposure to care tasks. In those sub-populations, potentially differing scoring cut-offs may be recommended.

We recommend users of the both the short and long-form scales to consider additional introductory language explaining why they are administering the tool, how it will be used in support of their children’s care, and that the results will be kept confidential. This language aligns with best practices in social determinants of health screening, in which family caregivers may be hesitant to share their experiences with food or housing insecurity [31, 32]. In this case of our self-efficacy managing health tasks, family caregivers may be hesitant to reveal their lack of confidence in providing their children’s medical care over concerns this may be interpreted as neglectful or potentially harmful. This is especially important given the disproportionate rate of referrals for neglect for children with medical complexity compared to other children [33, 34] and for groups that have experienced structural racism and/or other systematic discrimination like ableism [35, 36]. 

### General limitations and future work

This work is not without limitations. First, parent responses may have been susceptible to social-desirability bias because parents may have perceived it to be socially undesirable to admit to having challenges with any of the health caregiving activities for their child. Second, we did not have clinical information about the children in the household with which to examine associations between caregiver self-efficacy and clinical outcomes. This will be an important step for future work. Third, because not all parents received the subscales for self-efficacy for medication and self-efficacy for equipment, we opted to conduct three separate factor analyses, one for each subscale (self-efficacy for general care, self-efficacy for medication, and self-efficacy for equipment). We did this rather than conduct a single factor analysis with a three-factor structure. Although this is suitable in this circumstance, it also complicates interpretation. Fourth, our approach to item selection was data driven using psychometric evaluation. It is possible that if other approaches were used (e.g., item response theory), there may have been variation in the specific items that were retained for the short-form scales. Furthermore, we did not consult with parents and families to inform our final item selections. However, item generation for the original long-form scales were informed by parent focus groups and interviews, therefore the items retained on the short-form scales were developed with extensive parent input. We also had a relatively small Spanish language participant response that may have lost some differences from that population.

Next steps include examining temporal stability characteristics over varying time periods (e.g., test-retest reliability) [37]. Additionally, we intend to assess the predictive validity of the measure, for example, whether the level of self-efficacy of a parent managing their child’s care predicts outcomes like emergency department use, medication adherence, or symptom control.

Further, given that research is increasingly describing how family caregivers experience high levels of caregiver stress and a desire for increased supports in caring for their child at home [38, 39], further inquiry might assess whether interventions to enhance self-efficacy might reduce caregivers stress.

## Conclusion

This 13-item short form (5 general items, 3 equipment items, 3 medication items, 1 therapy item, 1 mobility item) is an efficient, self-administered means of assessing English and Spanish language family caregivers’ feelings of self-efficacy in their ability to manage the care of CYSHCN general, equipment, and medication healthcare needs. We additionally provided a means and standard deviations for identifying participants responding outside of national norms.

## Supplementary Information

Below is the link to the electronic supplementary material.

Supplementary Material 1

Supplementary Material 2

## Data Availability

The datasets generated and/or analyzed during the current study can be requested from the Children and Youth with Special Health Care Needs National Research Network primary data center, https://sprnetwork.org/resources-for-network-research/.
