# Supplementary material for: Short form scale of parent self-efficacy for general and specialized care for children with special health care needs
Source: J Patient Rep Outcomes. 2026 Jul 14;10:185. doi: 10.1186/s41687-026-01143-9 (PMC13650088; doi:10.1186/s41687-026-01143-9)
Supplement: Supplementary file 1 [file 41687_2026_1143_MOESM1_ESM.docx]

**Self-Efficacy Measure**

ENGLISH SHORT FORM PAPER VERSION

**Question 1.** Please respond to each question or statement by marking one box per row based on your **CURRENT level of CONFIDENCE.** Please answer based on your comfort with these tasks, even if your child may do some of these themselves.

|  | **(1) I am not confident at all** | **(2) I am a little confident** | **(3) I am somewhat confident** | **(4) I am quite confident** | **(5) I am very confident** |
| --- | --- | --- | --- | --- | --- |
| I can work with my child's doctor(s) to choose the treatment that seems right for my child, including the option of not giving any treatment. |  |  |  |  |  |
| I know when my child needs to be seen by a healthcare provider, if sick. |  |  |  |  |  |
| I know what to do if my child's misses a medication or other type of treatment. |  |  |  |  |  |
| I know the steps needed to schedule my child’s healthcare appointments. |  |  |  |  |  |
| I know what to do if my child has a medical emergency until help arrives. |  |  |  |  |  |

**Question 2. Does your child take any medications on a regular basis?**

- **Yes**
- **No**

**If your response to the above question is ‘Yes’, please fill out Question 3.**

**If your response to the above question is ‘No’, then continue onto Question 4.**

**Question 3.** Please respond to each question or statement by marking one box per row based on your **CURRENT level of CONFIDENCE.** Please answer based on your comfort with these tasks, even if your child may do some of these themselves.

|  | **(1) I am not confident at all** | **(2) I am a little confident** | **(3) I am somewhat confident** | **(4) I am quite confident** | **(5) I am very confident** |
| --- | --- | --- | --- | --- | --- |
| I know how to give my child's medications (such as by mouth or by tube). |  |  |  |  |  |
| I know how to arrange for my child to receive medication or treatments at locations other than home, if needed. |  |  |  |  |  |
| I can tell the difference between when my child is having a medication side effect or experiencing symptoms of their condition(s). |  |  |  |  |  |

**Question 4. Does your child use any medical equipment? Examples of medical equipment may items such as a feeding tube, pump, monitor, ventilator, or wheelchair.**

- **Yes**
- **No**

**If your response to the above question is ‘Yes’, please fill out Question 5.**

**If your response to the above question is ‘No’, then you are finished with this survey.**

**Question 5.** Please respond to each question or statement by marking one box per row based on your **CURRENT level of CONFIDENCE.** Please answer based on your comfort with these tasks, even if your child may do some of these themselves.

|  | **(1) I am not confident at all** | **(2) I am a little confident** | **(3) I am somewhat confident** | **(4) I am quite confident** | **(5) I am very confident** |
| --- | --- | --- | --- | --- | --- |
| I can change my child's disposable supplies or devices (such as diabetes pump, tracheostomy, line dressing, etc.). |  |  |  |  |  |
| I can tell when parts of my child's medical equipment, parts, or supplies needs to be replaced or repaired. |  |  |  |  |  |
| I know the settings on my child's medical equipment (such as a pump, monitor, ventilator, etc.). |  |  |  |  |  |

**We thank you for your time spent taking this survey.**
