# Supplementary material for: Short form scale of parent self-efficacy for general and specialized care for children with special health care needs
Source: J Patient Rep Outcomes. 2026 Jul 14;10:185. doi: 10.1186/s41687-026-01143-9 (PMC13650088; doi:10.1186/s41687-026-01143-9)
Supplement: Supplementary file 2 [file 41687_2026_1143_MOESM2_ESM.docx]

**Supplementary Figure 1:** Scree Plots for Exploratory Factor Analysis

Supplemental figure shows screen plots for exploratory factor analysis for the self-efficacy of general care (1a), medication care (1b), and equipment care (1c).

**c. Self-Efficacy for Equipment Care**

**a. Self-Efficacy for General Care**

**b. Self-Efficacy for Medication Care**
